# An overview of the impact of PFAS on animals, humans, and the environment using a One Health approach

**DOI:** 10.1007/s11356-026-37412-9

**Published:** 2026-01-24

**Authors:** Francesca Ferretti, Andrea Barbarossa, Anisa Bardhi

**Affiliations:** Department of Veterinary Medical Sciences, University of Bologna, Ozzano Dell’Emilia, Bologna, Italy

**Keywords:** PFAS, One Health, Toxicity, Bioaccumulation, Exposure, Emerging contaminants

## Abstract

Per- and polyfluoroalkyl substances (PFAS) are a group of synthetic chemicals characterized by a fluorinated carbon chain that confers unique physicochemical properties. Widely used in industrial and consumer products, including textiles, food packaging, and firefighting foams, PFAS are highly persistent in the environment, earning them the designation of “forever chemicals.” Their stability contributes to their widespread diffusion across different environmental compartments (water, soil, air) and multiple exposure pathways (e.g., diet). These lead to PFAS bioaccumulation and biomagnification, which poses a substantial threat to both ecosystems and human health. Exposure to PFAS has been associated with a range of adverse health effects, including liver damage, thyroid disease, immunotoxicity, reproductive issues, and various cancers in both humans and animals. While regulatory efforts have led to the phase-out of long-chain PFAS such as perfluorooctane sulfonate (PFOS) and perfluorooctanoic acid (PFOA), emerging research suggest that their short-chain replacements may also raise health concerns. This review applies a One Health framework to explore the interconnected impacts of these contaminants on human, animal, and environmental health. Furthermore, it highlights knowledge gaps that hinder comprehensive risk assessment and management, emphasizing the need for a globally coordinated, multidisciplinary approach to address the multifaceted challenges posed by PFAS.

## Introduction

Per- and polyfluoroalkyl substances (PFAS) are a class of commercially significant chemicals characterized by fully or partially fluorinated aliphatic chains of varying lengths that terminate in functional groups, such as sulfonate, carboxylate, or alcohol (Buck et al. 2011). Based on their chain length, PFAS are categorized as either short-chain or long-chain compounds (Table 1). Short-chain PFAS include perfluoroalkyl carboxylic acids (PFCAs) with seven or fewer carbons, such as perfluorohexanoic acid (PFHxA) and perfluorohexane sulfonic (PFHxS), as well as perfluoroalkyl sulfonic acids (PFSAs) with five or fewer carbons, such as perfluorobutane sulfonic acid (PFBS). Long-chain PFAS consist of PFCAs with eight or more carbons, such as perfluorooctanoic acid (PFOA), and PFSAs with six or more carbons, including perfluorooctane sulfonic acid (PFOS) (Peritore et al. 2023). PFAS are notable for their high thermal and chemical stability. This property is primarily attributed to the robust carbon–fluorine bonds due to their high bond energy (Brake et al. 2023a, b). The water- and oil-repellent properties of the perfluoroalkyl moiety have made these substances indispensable in numerous commercial and consumer applications. They are widely used in textiles, carpets, and leather treatments, as well as in surfactants, firefighting foams, and grease-resistant paper coatings (Buck et al. 2011).

**Table 1:** Classification of per- and poly-fluoroalkyl substances according to chain length and structural characteristics. The classification criterion distinguishes long-chain compounds (≥ C8 for perfluorocarboxylic acids and ≥ C6 for perfluorosulfonic acids), short-chain compounds (≤ C7 for perfluorocarboxylic acids and ≤ C5 for perfluorosulfonic acids), and emerging compounds, which include novel substances introduced as replacements for legacy chemicals

Classification	Abbreviation	Full name	Chemical formula	Molecular weight (g/mol)
Long-chain PFAS	PFOA	Perfluorooctanoic acid	C_8_HF_15_O_2_	414.07
	PFOS	Perfluorooctane Sulfonic acid	C_8_HF_17_SO_3_	500.13
	PFTrDA	Perfluorotridecanoic acid	C_13_HF_25_O_2_	664.10
	PFNA	Perfluoronanoic acid	C_9_HF_17_O_2_	464.08
	PFDA	Perfluorodecanoic acid	C_10_HF_19_O_2_	514.08
	PFUnDA	Perfluoroundecanoic acid	C_11_HF_21_O_2_	564.09
	PFDoA (or PFDoDA)	Perfluorododecanoic acid	C_12_HF_23_O_2_	614.10
Short-chain PFAS	PFHpA	Perfluoroheptanoic acid	C_7_HF_13_O_2_	364.06
	PFHxA	Perfluorohexanoic acid	C_6_HF_11_O_2_	314.05
	PFHxS	Perfluorohexane Sulfonic acid	C_6_HF_13_SO_3_	400.12
	PFBS	Perfluorobutane Sulfonic acid	C_4_HF_9_SO_3_	300.10
	PFBA	Perfluorobutanoic acid	C_4_HF_7_O_2_	214.04
	PFPeA	Perfluoropentanoic acid	C_5_HF_9_O_2_	264.05
Emerging PFAS	GenX (or HFPO-DA)	Hexafluoropropylene oxide dimer acid	C_6_HF_11_O_3_	330.05
	ADONA	Ammonium 4,8-dioxa-3H-perfluorononanoate	C_7_H_5_F_12_NO_4_	378.07
	F-53B	6:2 chlorinated polyfluoroalkyl ether sulfonate	C_8_HClF_16_O_4_S	532.58

However, this same stability that underpins their utility also presents significant environmental and health challenges. The extreme resistance of PFAS to thermal degradation, biodegradation, hydrolysis, and metabolization has led to their accumulation and persistence in the environment, since their production began in the late 1940 s (EFSA 2008; Armitage et al. 2006). Today, they are commonly referred to as “forever chemicals.” This term underscores the profound challenges posed by their persistent accumulation and the complexity of their removal from ecosystems (Jovanović et al. 2024; Göckener et al. 2023). Once released into the environment, PFAS and their degradation products present a serious threat. They bioaccumulate in living organisms and contaminate the food chain for decades (Jovanović et al. 2024). Biomonitoring studies have demonstrated the ubiquitous presence of PFAS in various biological matrices in both humans and animals, including plasma, serum, urine, feces, milk, and fur (Brase et al. 2021; EFSA 2020; Ma et al. 2020; Makowska et al. 2021; Barbarossa et al. 2013). These findings provided valuable insights into exposure pathways. Nonetheless, significant challenges remain in standardizing biomonitoring techniques and interpreting variability across species, regions, and trophic levels (Drew et al. 2021; Brase et al. 2021; Calafat et al. 2019; Verley et al. 2024; Jian et al. 2018).

Recognizing the environmental persistence and potential health risk of PFOA and PFOS, their gradual phase-out has driven the production of alternative PFAS compounds. These include short-chain homologs and novel fluorinated alternatives (reported in Table 1), such as perfluoro-2-propoxypropanoic acid (GENX), ammonium 4,8-dioxa-3H-perfluorononanoate (ADONA), C6O4 and 6:2 chlorinated polyfluoroalkyl ether sulfonic acid (6:2 ClPFESA; also known as F-53 B) (Pan et al. 2020; Birnbaum and Grandjean 2015). Initially, these alternatives were deemed safer due to their shorter chain lengths and presumed reduced stability; however, emerging evidence challenges this assumption. Short-chain PFAS are high mobility in soil and water, which allows them to rapidly contaminate drinking water resources. Their final degradation products also remain extremely persistent (Birnbaum and Grandjean 2015; Brendel et al. 2018; Munoz et al. 2019; Calafat et al. 2019; Pan et al. 2020; Fromme et al. 2017). Moreover, some compounds, such asF-53B, show greater bioaccumulative potential and longer half-lives in humans (up to 15.3 years) compared to PFOS. This raises concerns about their long-term impacts (Pan et al. 2020).

Concerning is the contamination of remote areas, far from industrial PFAS production sites (Butt et al. 2010; Muir et al. 2019; Valsecchi et al. 2021). The detection of PFAS in Arctic polar bears and other wildlife in isolated regions highlights the global transport of these chemicals through atmospheric and aquatic pathways (Giesy and Kannan 2001; Shoeib et al. 2006; Taniyasu et al. 2013; Yamashita et al. 2005; Ahrens and Bundschuh 2014). Throughout their lifecycle, from production to disposal, PFAS are released into the environment at multiple stages, with a tendency to migrate to oceans and marine sediments over time. Their widespread distribution and environmental persistence have prompted regulatory actions, including the phase-out of long-chain legacy PFCAs and sulfonic acids PFSAs in North America and Europe since the 2000 s (Evich et al. 2022).

Despite ongoing efforts, significant challenges remain. The replacement of legacy PFAS with short-chain alternatives poses concerns, as their environmental and health impacts are not yet fully understood. Furthermore, regulatory measures vary widely across regions. Many countries continue to produce and use PFAS under insufficient oversight. This review aims to synthesize the current literature on the impacts of PFAS on humans, animals, and the environment from a One Health perspective, examining the interconnected effects of these substances on this triad. One Health represents an integrated and multidimensional approach aimed at sustainably balancing and optimizing the health of humans, animals, and ecosystems. This paradigm is grounded in the recognition that human, animal, and environmental health are closely interconnected and mutually dependent. Within this framework, cross-disciplinary and cross-sectoral collaboration is essential to prevent, detect, and address emerging health challenges that transcend species and geographical boundaries. The adoption of the One Health model not only strengthens global health security but also contributes to achieving the Sustainable Development Goals, fostering a holistic and resilient vision of health. Finally, this review seeks to highlight existing knowledge gaps, assess the limitations of current mitigation strategies, and the pressing need for a globally coordinated response to address the multifaceted risks posed by PFAS within the One Health framework.

## PFAS persistence in the environment

PFAS can be released into the environment during production, use and disposal (Ahrens and Bundschuh 2014). They enter in the water cycle either directly through nonpoint sources, such as runoff and groundwater infiltration. They also originate from point sources, including firefighting training grounds, industrial facilities, municipal and industrial wastewater treatment plant effluent, and even through atmospheric deposition (Hu et al. 2016; Lu et al. 2017). Additional sources include air emissions, subsequent atmospheric deposition to soils, and leaching processes. PFAS can also volatilize from household products. They accumulate in house dust at concentrations detected in the nanogram-per-gram range (ng/g), as shown in several studies (Brunn et al. 2023; Strynar and Lindstrom 2008; Björklund et al. 2009; Beesoon et al. 2012). The fluorinated alkyl tail and polar head group of ionic PFAS strongly influence their environmental distribution. Partitioning properties and electrostatic interactions play a major role in this process. Their hydrophilic head imparts high water solubility, allowing PFAS to interact with and disperse in water (Environment and Climate Change Canada 2023). Additionally, PFAS tend to accumulate at the air-water interface as a result of their surfactant-like properties. Their hydrophilic head group dissolves in water, while the hydrophobic tail orients itself toward the air, leading to retention in the unsaturated zone (Costanza et al. 2019). Furthermore, transport to the deep ocean and sediment burial are considered to be environmental sinks for PFAS, given their extreme persistence in the environment (Prevedouros et al. 2006). Despite their widespread presence, the mechanisms governing PFAS transport and environmental fate remain an active area of research (Verley et al. 2024). Understanding these processes is essential for effectively managing and mitigating PFAS contamination. However, their extreme persistence and multiple pathways of spread pose significant challenges for remediation and raise serious concerns about long-term implications for ecosystems and human health.

### Water

PFAS can enter water systems through numerous pathways, creating significant environmental and health concerns. Runoff from contaminated sites -including manufacturing plants, fields treated with biosolids, and landfills- can flow directly into lakes and rivers or infiltrate into the ground, contaminating wells and aquafers (Bräunig et al. 2017; Bolan et al. 2021). Atmospheric transport worsens the issue as clouds over PFAS-contaminated sites can carry contaminants hundreds of kilometers away from the source (Sammut et al. 2017). Ocean currents further contribute to the global spread of PFAS, transferring contamination to distant regions (Muir and Miaz 2021).

Natural waters, which serve as habitats for various aquatic species, accumulate PFAS leading to bioaccumulation and biomagnification (Xing et al. 2023). Fish in polluted ecosystems are known to accumulate PFAS, posing risks to both ecological systems and human food safety (Goodrow et al. 2020). Similarly, livestock, such as cattle and sheep, can ingest PFAS through contaminated water, resulting in PFAS-contaminated meat and dairy products (Drew et al. 2021). Furthermore, PFAS contamination in domestic water supplies represents a direct exposure route for humans and domestic animals (Borrull et al. 2021).

### Air

Atmospheric PFAS sources include emissions from industrial processes, wastewater treatment facilities and landfills (Ahrens et al. 2011). PFAS concentrations near these locations are 5–30 times higher than in other areas (Xing et al. 2023). Therefore PFAS can travel over long distances through airborne particulate matter (Death et al. 2021) and are found in the atmosphere not only as legacy compounds such as PFOA and PFOS but also as short-chain substances like perfluorobutanoic acid (PFBA) and PFHxA (Yamazaki et al. 2021). Both humans and animals are exposed to PFAS in the air through inhalation or deposition on feathers and skin, particularly those in regions close to contaminated sites. This pathway is especially concerning for individuals living close to industrial facilities or landfill areas, with occupational exposure posing additional risks. A review on occupational PFAS exposure highlights that workers in certain occupations (e.g., professional ski waxers, firefighters, fluorochemical plant workers) experience high PFAS serum concentrations (Lucas et al. 2023). Ski waxers, for instance, are exposed through the inhalation of emissions during ski wax application to enhance ski performance, while firefighters are exposed to PFOS and PFHxS through the frequent use of aqueous film forming foam (AFFF) (Freberg et al. 2010).

### Dust

Dust is a complex mixture of aerosol particles and other biological materials, serving as a reservoir for numerous chemicals present in consumer products (Lucattini et al. 2018). These chemicals can accumulate in dust through off-gassing or abrasion from treated materials and consumer products (Lin et al. 2022; Savvaides et al. 2021). Given that people spend approximately 80–90% of their time indoors, making indoor environments a major source exposure (Slezakova et al. 2012; Šujanová et al. 2019). Moreover, PFAS concentration in indoor air can be up to 100 times higher than those measured outdoors (Shoeib et al. 2004).The combined effects of treated materials, dust accumulation, and behavioral tendencies create a unique vulnerability for children and pets (Kotthoff et al. 2015; Bost et al. 2016).

Crawling on PFAS-treated carpets and stuff, combined with frequent hand-to-mouth activities, raises the probability of toddlers ingesting PFAS-contaminated dust (Trudel et al. 2008). Similarly, pets, due to their close contact with dust-collecting areas, may experience analogous exposure levels (Brake et al. 2023a, b).

### Soil

Soil acts as a major reservoir for PFAS. These compounds accumulate through rainfall, runoff, irrigation, and the application of biosolids and pesticides recycled from urban wastewater treatment. Key sources of PFAS soil pollution include industrial processes, waste leachate, occupational waste, and firefighting activities (Navarro et al. 2017; Nascimento et al. 2018; Abou-Khalil et al. 2022; Zhu et al. 2022). Animals can ingest PFAS from contaminated soil while feeding or digging and from plants grown in this ground. They can also be exposed through inhalation when these compounds become airborne, leach into groundwater, or run off into surface water (Brake et al. 2023a, b). Farmland contamination particularly raises risks for PFAS accumulation in livestock and poultry, impacting food safety (Xing et al. 2023; Sharp et al. 2021).

**Fig. 1:**
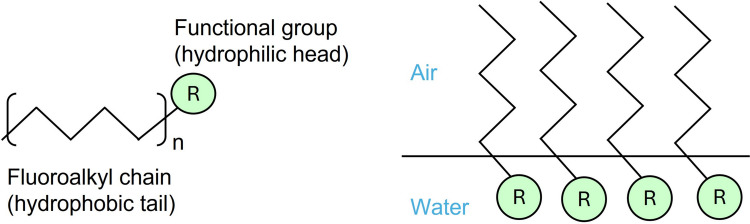
Chemical structure of per- and poly-fluoroalkyl substances, highlighting features responsible for their environmental persistence. Strong carbon–fluorine (C–F) bonds confer high chemical stability, while the hydrophobic fluorinated chain (R groups) influences behavior in air and water, contributing to widespread distribution and resistance to degradation

To highlight the chemical features responsible for the environmental persistence of PFAS, we have included Fig. 1. This figure summarizes key structural characteristics, such as the strong C–F bonds and hydrophobic–lipophobic properties, which contribute to their stability in water and air and complicate their degradation.

## PFAS exposure pathways for humans and animals

**Fig. 2:**
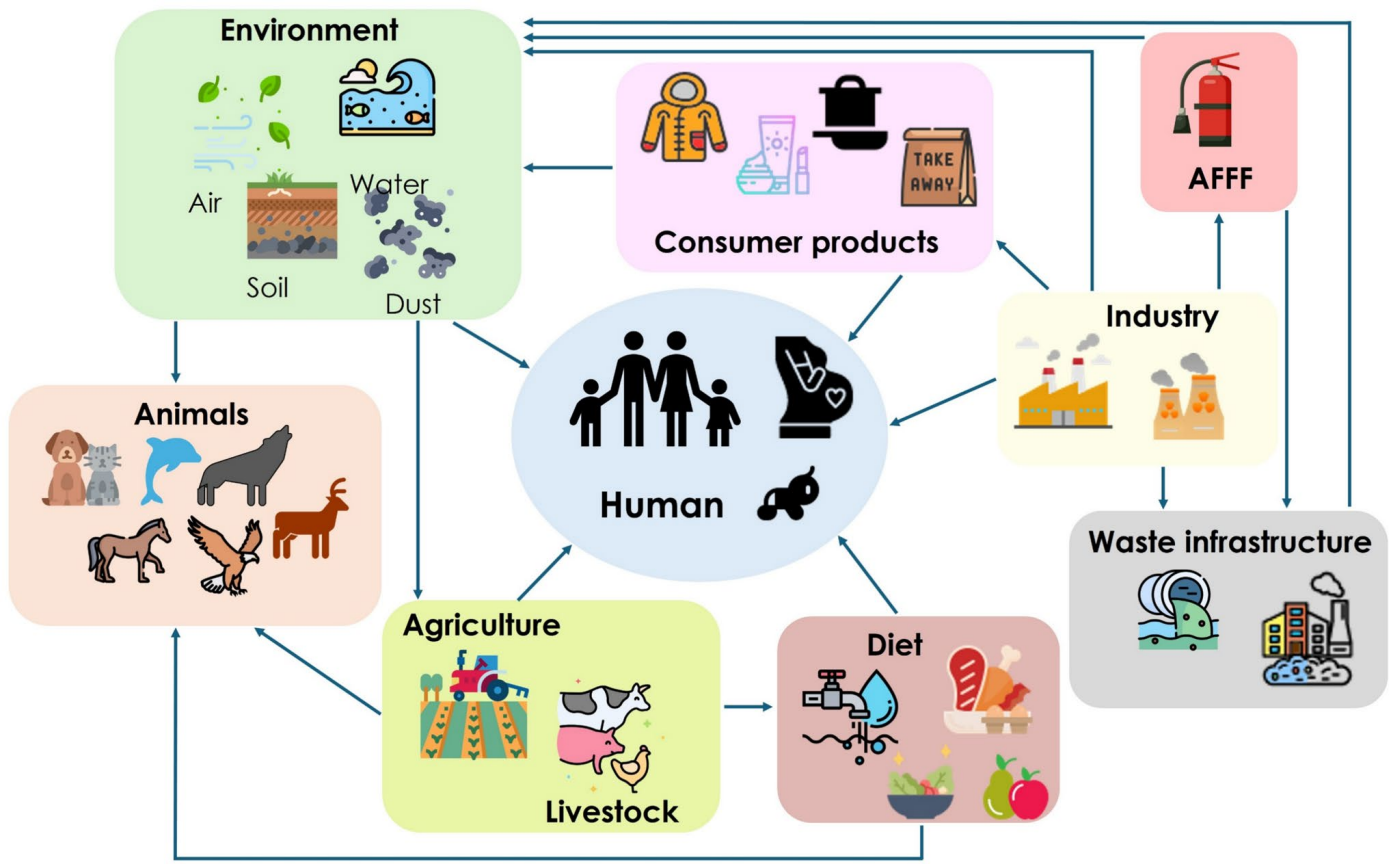
Per- and polyfluoroalkyl substances exposure pathways

Once PFAS are released they settle in the surrounding environment, contaminating all surfaces (Dauchy 2023). Due to their resistance to biological degradation, both humans and animals can be exposed to these chemicals through multiple direct and indirect pathways (Jha et al. 2021). These include both those derived from the environmental pathways described in the previous section and the major categories listed below: exposure through diet, dermal absorption from contaminated products or dust, and maternal transfer (Fig. 2).

### Diet

Dietary intake is a major pathway for PFAS exposure in humans. The European Food Safety Authority (EFSA) determined that dietary PFAS intake in adults is largely attributed to fish and other seafood, which can account for up to 86% of their exposure (EFSA 2018). This contamination arises from consuming agricultural products that absorb PFAS from soil and water, as well as animals and their products (e.g., eggs and milk). Exposure can occur via air, soil and through the ingestion of contaminated feed and water, underscoring the potential for PFAS bioaccumulation across the food chain (Xing et al. 2023).

Food packaging is another contributor to dietary PFAS exposure. Material such as containers, wraps, kitchenware coatings, and disposable paper cups can contain PFAS that leach into food during storage and consumption (Zabaleta et al. 2020). Studies have shown cases of PFAS concentrations in food packaging, reaching ng/L levels (Zabaleta et al. 2020). PFAS migration is more pronounced in high-fat foods and at elevated temperatures, with longer-chain PFAS exhibiting greater adsorption by fats, whereas short-chain PFAS tend to volatilize (Elizalde et al. 2018). Although PFAS in food contact materials contribute to human exposure, research indicates that this route represents a smaller fraction of the total dietary exposure compared to other sources (EFSA 2020).

### Dermal absorption

Dermal exposure to PFAS can occur through contact with house dust and various consumer products. These include carpets, textiles such as technical clothing, waxes and paints, food packaging, non-stick cookware and personal care items like cosmetics (EFSA 2020). Research into the dermal absorption of PFOA has been conducted utilizing isolated epidermal preparations from rodents and humans (Fasano et al. 2005; Franko et al. 2012). These studies suggest that dermal absorption depends on the ionization state of PFOA, with a significant dermal absorption potential when the compound is in its unionized form. However, under environmental exposure conditions, PFOA primarily exists in its ionized form, which considerably reduces its ability to penetrate human skin (Franko et al. 2012). This implies that while significant dermal absorption of PFOA may occur in laboratory settings, it is unlikely to be a major route of exposure in environmental scenarios. Moreover, the findings indicate that rat and mouse skin is more permeable to PFOA compared to human skin, raising questions about the relevance of animal models in accurately assessing human dermal exposure risk. In contrast, a recent study assessed human dermal absorption of 17 PFAS using in vitro 3D human skin equivalent models exposed to 500 ng/cm^2^ PFAS dissolved in methanol over 24–36 h. The researchers found that short-chain PFAS are readily absorbed through the skin. Perfluoropentanoic acid (PFPeA) and PFBS had the highest absorbed fraction, 58.9% and 48.7% respectively. The absorbed fraction decreased with increasing carbon chain length, highlighting the potential significance of dermal exposure as a pathway for human short-chain PFAS accumulation (Ragnarsdóttir et al. 2024).

These studies highlight the complexity of assessing dermal exposure to PFAS, which appears to be limited under typical environmental conditions. However, further research is needed to evaluate other PFAS compounds and their potential impact, particularly given their widespread presence in consumer products and dust.

#### Maternal transfer

PFAS are capable of crossing the placental barrier in humans, resulting in fetal exposure during critical developmental stages. Additionally, infants can be exposed to PFAS through breastfeeding, as these chemicals are transferred via lactation (Ricolfi et al. 2024). A study specifically highlighted lactation as a source of PFOS and PFOA exposure for newborns. First-born children may be at higher risk due to the life time accumulation of these chemicals in the maternal body. It has also been suggested that a long interval between pregnancies may allow maternal PFAS levels to rise again. This could increase exposure risks for subsequent offspring (Barbarossa et al. 2013). Maternal transfer of PFAS is not limited to humans. Similar patterns of PFAS transfer have been documented in aquatic mammals, reptiles, fish, and birds (Ricolfi et al. 2024). Evidence of PFAS secretion through milk has also been observed in livestock, with studies confirming its presence in cattle milk (Kowalczyk et al. 2013).

These findings underscore the far-reaching implications of maternal PFAS transfer across species. While lactation is a well-documented exposure pathway, the bioaccumulation and persistence of PFAS in maternal tissues highlight the need for further investigation into intergenerational risks. Given its potential to impact vulnerable populations such as newborns and wildlife, regulatory actions are critical to reducing PFAS contamination in the environment.

## Species-specific exposure

Exposure pathways and sources are not uniform across humans and the different animal species, but are shaped by their ecological niches, behaviors, and interactions with anthropic activities. To capture these differences, it can be useful to classify exposure into “species-specific” categories, distinguishing among humans, wildlife, and companion animals. This approach highlights both common exposure routes and unique risk factors that are critical for interpreting PFAS distribution and potential health effects*.*

### Humans

For humans, the main pathways of PFAS exposure are through food, drinking water, and consumer products. However, individuals living near sites with historical PFAS contamination, such as military bases, airports, or wastewater treatment plants handling industrial waste, are at greater risk of elevated exposure levels (De Silva et al. 2021). Occupational exposure to PFAS primarily occurs via inhalation and skin contact (Franko et al. 2012). Inhalation is especially concerning in workplaces where volatile PFAS intermediates vaporize during manufacturing processes and subsequently hydrolyze into stable PFAS compounds (Kaiser et al. 2010). One notable example is professional ski wax technicians, who exhibit higher blood levels of PFCA, with concentrations increasing in proportion to years of employment. Ski wax formulations often contain both precursor polyfluorinated compounds and PFCAs. When applied at high temperatures (130–220 °C), they release gaseous substances and particles (Carlson and Tupper 2020).

These findings highlight the importance of occupational environments as significant contributors to PFAS exposure, particularly in industries that involve heating or processing materials containing these chemicals.

### Wildlife

Higher PFAS exposure in wildlife raises critical concerns for both animal health and human populations that rely on these species as food sources. Wild animals encounter PFAS through polluted environments such as contaminated water bodies, dietary intake and contact with tainted soil and air (Andrews et al. 2023). The bioaccumulation of these chemicals not only threatens ecological balance but also compromises food safety, emphasizing the urgent need for holistic strategies to manage PFAS pollution across ecosystems.

### Pets

High PFAS concentrations in indoor environments contribute significantly in PFAS exposure in both humans and pets as they often share common exposure sources (e.g., food, drinking water, dust, and air) (Kotthoff et al. 2015; Death et al. 2021; Brake et al. 2023a, b). In addition, textiles produced for pets and children can contain PFAS due to the widespread use of PFAS-based stain-resistant treatments in fabrics and plastics (Glüge et al. 2020). PFAS are also well-documented in human food packaging as well as in pet food and its packaging (Schaider et al. 2017; Chinthakindi et al. 2021).

## Bioaccumulation of PFAS in different species

The bioaccumulation nature of PFAS raises significant concerns about potential health risks to humans, pets, and wildlife (Jha et al. 2021; Dimitrakopoulou et al. 2024). Upon ingestion, PFAS are almost completely absorbed through the gastrointestinal tract. They can accumulate in various tissues, primarily serum, liver, and kidneys, due to their affinity for hepatic and plasma proteins (Brake et al. 2023a, b). Research has shown that PFAS bioaccumulate in many animal species, with apex predators at greatest risk (Chen et al. 2021; Wu et al. 2020; Fair and Houde 2018). Seabirds, marine mammals, and terrestrial species exhibit the highest magnification factors. In contrast, organisms in exclusively aquatic food webs, such as fish with gills, are more efficient at eliminating perfluoroalkyl acids (De Silva et al. 2021). In most livestock and poultry species, PFAS concentrations are consistently higher in the liver than in muscle tissues (Guruge et al. 2016). This mirrors the distribution characteristics observed in fish, indicating that the liver tissue affinity and precursor transformation ability for PFAS were independent of species (Shi et al. 2018). In contrast to bioaccumulation in animals, plants show a preference for accumulating short-chain over long-chain PFAS (Brunn et al. 2023).

The bioaccumulation efficiency is influenced by metabolic half-life of PFAS in organisms. This reflects tissue- and organ-specific affinities or metabolic activity toward particular functional groups (Death et al. 2021). In both humans and animals, shorter-chain PFAS compounds are more water-soluble, less likely to bind to plasma proteins, and thus eliminated more quickly than longer-chain compounds (Gomis et al. 2018). PFAS are primarily excreted through urine or feces, although elimination rates and half-lives vary depending on the chemical and the animal species (Gomis et al. 2018; Death et al. 2021). For instance, the half-life of PFOS in mice spans from several days to weeks, while in pigs, it can remain for up 634 days (Brake et al. 2023a, b). The half-life of PFOA in mice can be as short as 2 h, while in pigs, it can reach 236 days (Numata et al. 2014). In dogs, PFOA's half-life varies from 8 to 30 days (Brake et al. 2023a, b). In humans, the serum half-lives of PFOS, PFHxS, and PFOA are approximately 4.77, 5.35, and 2.35 years, respectively (Rosato et al. 2024b).

## Associations of PFAS with different disease in humans and animals

Several epidemiological studies have explored the health effects of PFAS exposure, and their findings have been reviewed in this section. These investigations revealed associations with various human health outcomes, including immune and thyroid alterations, metabolic dysfunction, liver and endocrine disorders, adverse reproductive and developmental outcomes, and cancers (Bonato et al. 2020; Fenton et al. 2021). Similar to humans, wild and domestic animals exposed to PFAS have exhibited various adverse health effects. Evidence increasingly suggests that elevated PFAS exposure coincides with the rise in pet diseases, including conditions analogous to those observed in humans such as cancer, thyroid disorders, diabetes, reproductive failure, as well as heart and kidney diseases (Wang et al. 2019; Brake et al. 2023a, b). It is important to note that responses to PFAS can vary significantly across species due to differences in metabolism, physiology, and ecological niches. While animal studies provide critical mechanistic insights, direct extrapolation to humans should consider these interspecies differences. This section aims to provide a summary of toxic effects related to PFAS exposure, first focusing on alterations linked to human health and then on animal diseases.

### Humans

#### Hepatic effects

Studies on PFOA, PFOS, and PFHxS have documented elevated liver enzyme levels and reduced serum bilirubin, suggesting potential liver alterations (ATSDR 2021). An Italian study examined the relationship between exposure to several PFAS and levels of alanine transaminase (ALT) and aspartate transaminase (AST), biomarkers of liver dysfunction. The study involved adults aged 20 years and older living in the highly PFAS-contaminated Veneto Region, in Italy. Scientists found a correlation between higher PFOA concentrations and increased ALT, especially in men. Associations with PFHxS and perfluorononanoic acid (PFNA) levels were also observed in both sexes (Rosato et al. 2024a).

### Cardiovascular effects

De Toni et al. investigated the impact of PFOA exposure on platelet function, which plays a key role in atherosclerosis acute thrombotic events (De Toni et al. 2020). This study evaluated PFOA uptake in platelets, alterations in platelet membrane fluidity, and platelet activation in response to different PFOA doses in 48 men living in a high-PFAS-exposure area in Veneto. The major target of PFOA were the platelet membranes with dose-dependent accumulation increasing membrane fluidity. Exposed subjects exhibited higher serum and platelet levels of PFOA, along with increased platelet aggregation levels, compared to controls. The presence of PFOA was associated with increased cytosolic calcium concentration in platelets.

Recently, a large body of epidemiological data, reviewed by Wen et al. suggests that exposure to both legacy and emerging PFAS in serum/plasma may be linked to adverse cardiovascular outcomes. Reported associations include congenital heart disease, coronary artery disease, cardiometabolic risk, cardiac structural change, atherosclerosis, stroke, sex-specific hypertension (Wen et al. 2023; Averina et al. 2021; Birukov et al. 2021; Huang et al. 2018; Feng et al. 2022a; Manzano-Salgado et al. 2017; Pitter et al. 2020). These studies also highlight numerous in vivo and in vitro experiments showing that PFAS exposure adversely affects cardiovascular health. Reported effects include cardiac dysfunction, morphological abnormalities, cardiomyocyte apoptosis and mitochondrial damage, disturbance of myocardial differentiation and vascular formation, impairment of endothelial cell junctions and specialized vascular barriers, hypertension in offspring, and abnormal blood lipid (Wen et al. 2023). These results contribute to understanding the growing link between PFAS exposure and cardiovascular diseases.

### Developmental effects

PFAS exposure has been linked to altered fetal and postnatal growth, including reduced birth weight (Liew et al. 2018). Evidence suggests a correlation between serum levels of PFOA and PFOS and small decreases in birth weight, with a decrease of < 20 g for each 1 ng/mL increment in blood PFOA or PFOS level (ATSDR 2021).

### Thyroid dysfunctions

Emerging PFAS have been shown to disrupt thyroid hormone production, leading to abnormal thyroid function in adults, adolescents and also pregnant women. Specifically, PFAS exposure was linked to decreased levels of thyroid-stimulating hormone (TSH) and elevated levels of free thyroxine (FT4) and triiodothyronine (T3). These alterations may cause hyperthyroidism and disrupt endocrine and immune functions (Du et al. 2024). In adolescent males, a positive association was observed between exposure to certain PFAS and TSH levels (Ballesteros et al. 2017) . Among men over the age of 50, PFAS exposure was linked to decreased levels of TSH and FT4, alongside elevated levels of thyroxine (T4) and T3. Additionally, PFAS exposure may harm the thyroid gland in older populations (Tan et al. 2024). A recent in vitro study compared the thyroid disrupting effects of PFOA and its alternatives, GenX and ADONA, on rat and human thyroid cells. The findings revealed that the disruptive impact increased in the order of GenX > PFOA > ADONA (Zhang et al. 2021). Furthermore, several studies have suggested a relationship between PFAS exposure and thyroid hormone alterations in pregnant women, raising concerns for both maternal and fetal health (Du et al. 2024; Zheng et al. 2024). Meanwhile, PFAS can pass from mother to fetus through the placental barrier, leading to in utero exposure) that may have a negative impact on the birth size and thyroid function of the offspring (McAdam and Bell 2023; Xiao et al. 2020).

### Cholesterol

PFAS exposure may also alter metabolic functions, particularly cholesterol levels. These substances may bindto PPARs (peroxisome proliferator-activated receptors) and other nuclear receptors, raising concerns about their potential impact on cholesterol levels (Bjork et al. 2011). Several studies have explored the associations between PFAS and cholesterol levels, though findings are largely inconsistent. The exception is PFNA, for which studies have found associations with total cholesterol (Graber et al. 2018; Seo et al. 2018; Fu et al. 2014; Zeng et al. 2015; Jain and Ducatman 2019; Lin et al. 2019; Nelson et al. 2019). Although PFNA often shows moderate correlation with PFOS and PFOA, it is challenging to separate its specific effects from those of PFOS and PFOA. This is especially true in smaller studies. Nevertheless, the data suggest that PFNA’s association with serum cholesterol is independent of PFOS and PFOA (EFSA 2020).

### Diabetes

Emerging evidence indicates that PFAS can act as endocrine disruptors and may contribute to the development of type 2 diabetes (T2D). In a study by Sun et al., researchers examined associations between PFAS exposures and the later occurrence of T2D. They also evaluated demographic factors and lifestyle that influence PFAS concentrations in plasma (Sun et al. 2018). Even after accounting for established T2D risk factors, including body mass index, family history, physical activity, and other variables, the study revealed that higher plasma concentrations of PFOS and PFOA were associated with an increased risk of T2D. These results suggest that exposure to PFAS, especially PFOS and PFOA, may play a role in the development of diabetes. Additionally, PFAS may have stronger effects in individuals already at risk for diabetes (e.g., overweight) or during critical periods of weight change, such as childhood growth spurts and puberty (Timmermann et al. 2014; Domazet et al. 2016). Further studies are needed to determine whether the effects of PFAS exposure are age and/or sex dependent and to elucidate the mechanisms underlying these associations which remain unclear.

### Reproductive effects

PFAS have been shown to contribute to female infertility enhancing reactive oxygen species (ROS) production, compromising progesterone production, and interfering with its hormonal activity (Fenton et al. 2021). Specifically, PFOA demonstrates a consistent binding affinity for progesterone, disrupting the activation of progesterone-related genes in endometrial cells (Di Nisio et al. 2020). Experimental and epidemiologic evidence indicates that PFAS interfere with endocrine processes. They also exhibit reproductive and developmental toxicities. Polycystic ovary syndrome (PCOS) is a frequent hormonal disorder affecting women of reproductive age.Its a key factor to female infertility. Wang et al. conducted a case–control study evaluating associations between plasma PFAS levels and PCOS-related infertility (Wang et al. 2019). They found that elevated plasma concentrations of perfluorododecanoic acid (PFDoA) substantially raised the risk of PCOS-related infertility. Another study reported relevant dysregulation in the genetic cascade involved in embryo implantation and endometrial receptivity, with results pointing to hormonal interference by PFOA (Di Nisio and Foresta 2019). This is further supported by a significant delay of puberty and increased irregular menstrual cycles in young women living in highly exposed areas. In men, evidence regarding PFAS exposure and semen quality or levels of reproductive hormones is limited. Nevertheless, PFOA exposure has been associated with impaired sperm motility and reduced sperm penetration in viscous media (Yuan et al. 2020; Šabović et al. 2020). It is also correlated with reduced sperm concentration and total sperm count, coupled with increased levels of luteinizing (LH) and follicle stimulating (FSH) hormones in young men (Joensen et al. 2009; Vested et al. 2013; Song et al. 2018). Notably, in utero exposure to PFOA has been linked to lower sperm concentration and total sperm count, as well as elevated LH and FSH later in adulthood (Geueke 2016).

### Cancer risk

In November 2023, the International Agency for Research on Cancer classified PFOA as a Group 1 carcinogen, indicating it cause cancer in humans. PFOS was classified as Group 2B carcinogen, meaning it is possibly carcinogenic to humans. These classifications were based on a combination of experimental animal studies, mechanistic evidence, and limited human data (Zahm et al. 2024). PFAS exposure has been linked to various cancers, including testicular, breast, kidney, thyroid, and bladder tumors. Mechanisms such as hormone regulation, oxidative stress, and DNA damage are believed to contribute to carcinogenesis (Zheng et al. 2024).

Epidemiological studies have found positive associations between PFOA and PFOS exposure and an increased risk of breast cancer (Mancini et al. 2020; Li et al. 2022b). For example, higher levels of short-chain PFAS like perfluoroheptanoic acid (PFHpA) were linked to an increased risk of breast cancer in a Chinese study (Feng et al. 2022b). Similarly, elevated levels of perfluorodecanoic acid (PFDoA), perfluorodecanoic acid (PFDA), and perfluorohexanoic acid (PFHxA) were associated with breast cancer incidence in Filipino patients (Velarde et al. 2022).

Testicular cancer has one of the strongest links to PFAS exposure. A study of a community exposed to PFAS between 1950 to 2004 revealed that individuals with higher PFOA levels had a threefold increased risk of testicular cancer (Barry et al. 2013). Testicular germ cell tumors (TGCTs) are also among the eight cancers more frequently observed in PFAS exposed firefighters compared the general population (Soteriades et al. 2019; Boyd et al. 2022).

The kidney is particularly vulnerable to PFAS due to its role in excretion. PFAS may harm kidneys by being reabsorbed in renal tubules through efflux transporters, which prolong their half-life in human body (Blake et al. 2018; Han et al. 2012). Several studies indicate that PFAS exposure alters pathways linked to kidney diseases, including those involving oxidative stress and peroxisome proliferators—activated receptor, reduced kidney function, and increased mortality from kidney cancer (Bonato et al. 2020; He et al. 2023; Liu et al. 2023; Niu et al. 2023; Li et al. 2022c; Stevenson et al. 2021; Zheng et al. 2024). For instance, a Swedish cohort study found higher kidney cancer rates among individuals exposed to contaminated drinking water compared to controls (Li et al. 2022a). Similar findings were observed in other researches, including a study in the USA with background exposure levels (Shearer et al. 2021), an ecological mortality study in an area of Italy with PFOA contamination, and a PFOA occupational cohort mortality study (Steenland and Woskie 2012; Mastrantonio et al. 2018).

Recent research has identified PFOS as a notable risk factor for liver cancer. A case–control study demonstrated that individuals with elevated PFOS levels were 4.5 times more likely to develop hepatocellular carcinoma (Goodrich et al. 2022). A study conducted in China revealed significantly higher concentrations of PFOA, perfluorotridecanoic acid (PFTrDA), and PFBS in liver tumor tissues compared to healthy liver tissues (Liu et al. 2021). Additionally, it was observed that PFAS levels were generally lower in females than males, potentially due to biological differences in excretion mechanisms such as childbirth and breastfeeding (You et al. 2024).

The potential connection between PFAS exposure and thyroid cancer has become a worldwide concern due to the pervasive presence of these chemicals in the environment (Zheng et al. 2024). A cohort study analyzed plasma samples from 88 thyroid cancer patients and 88 matched healthy controls, accounting for variables such as sex, age, ethnicity, body mass index, smoking status, and sample collection year. The study found that an increase in plasma PFOS levels correlated with a higher risk of thyroid cancer. This association was especially strong in a subgroup of 31 patients diagnosed at least 1 year after sample collection (Van Gerwen et al. 2023). However, a recent meta-analysis synthesizing data from five human studies, including the aforementioned research, on PFOS, PFOA, PFNA, and PFHxS exposure found no clear link to thyroid cancer risk (Van Gerwen et al. 2023; Liu et al. 2022a; Cathey et al. 2023; Li et al. 2023; Madrigal et al. 2024). The review highlighted both the limited understanding of PFAS-related thyroid carcinogenesis and the variability in methodologies and outcomes across existing studies (Van Gerwen et al. 2024). Regarding bladder cancer, studies reported modest but not statistically significant associations between PFOA exposure and bladder cancer, while a Danish cohort study found no link between PFOA plasma levels and an increased risk of developing bladder cancer (Eriksen et al. 2009).

Messmer et al. examined cancer risks in the Merrimack, New Hampshire, community which was exposed to PFAS through drinking water and air contamination from a local factory (Messmer et al. 2022). Merrimack residents had a 47% higher risk of thyroid cancer compared to the general US population and a 69% higher risk compared to residents of four demographically unexposed towns. Between 2005 and 2014, residents also experienced significantly higher rates of mesothelioma and esophageal, thyroid, and bladder cancers. Specifically, there was a 45% increased risk of bladder cancer, a 71% increased risk of esophageal cancer, and a 141% increased risk of mesothelioma compared to the unexposed New England towns (Messmer et al. 2022).

### Immune effects

PFAS can disrupt immune function, adversely affecting immune organs, cells, and signaling molecules (Liang et al. 2022). Epidemiological data reveal correlations between elevated PFAS levels and heightened leukemia risk in both pediatric and adult populations (Jones et al. 2024; Winquist et al. 2023). Studies in diverse exposed populations have linked PFAS exposure to reduced vaccine efficacy against multiple antigens. They also show increased susceptibility to infections (Grandjean et al. 2012; Dalsager et al. 2021). These outcomes are corroborated by observed declines in cytokine production and weakened lymphoproliferative activity in human primary cells (Zahm et al. 2024). Further investigations highlight that maternal PFOS exposure is linked to higher risks of infectious disease-related hospitalizations in offspring. PFOS and PFOA exposure are also associated with hospitalizations for lower respiratory tract infections. These findings suggest PFAS may impair immune system development, predisposing individuals to both mild and severe infections (Dalsager et al. 2021).

### Animals

#### Endocrine and metabolic effects

Exposure to PFAS has been found to affect reproduction, growth, mobility, and survival of fish and aquatic organisms. For example, PFAS can induce oxidative stress and alter the regulation of genes and nuclear receptors involved in xenobiotic, lipid, and carbohydrate metabolism in fish (Lee et al. 2020). Effects on the endocrine and reproductive system have also been reported. Researchers found that chronic PFNA exposure in zebrafish can lead to dysfunction in the hypothalamic pituitary gonadal liver axis. It also disrupts sex hormone synthesisand decreases the gonadosomatic index (a measure of sexual maturity) and fertility (Zhang et al. 2016). Additionally, multigenerational studies have observed that PFAS exposure can affect mortality, fecundity, gonad development, and swimming rate in fish offspring and disrupt the thyroid endocrine system (Ji et al. 2008; Wang et al. 2011; Lee et al. 2017; Chen et al. 2018). In a study conducted on male guppies (*Poecilia reticulata)*, a popular freshwater fish species, negative effects on reproduction were seen with both legacy PFAS (PFOA) and a newly emerging PFAS (GenX). Fish exposed to GenX showed changes in male mating tactics and decreased sperm velocity. While PFOA bioconcentrated in fish at higher levels than GenX, the effect of PFOA on the reproductive characteristics described in the study was lower, with no significant effect on male sexual behavior despite similar reduction in sperm performance. These results suggest that emerging PFAS could be more harmful than the legacy compounds, even at environmentally realistic concentrations and with short-term exposure. Additionally, some genes involved in immune regulation, spermatogenesis, and sexual differentiation were modulated. Changes in spermatogenesis gene expression may indicate early impairment of sperm production. These findings suggest that reproductive phenotypes and gene expression patterns are sensitive endpoints for assessing PFAS toxicity in guppies, even at environmentally pertinent concentrations (Gasparini et al. 2024). PFAS has also been shown to impact the growth and development of early amphibian life stages (Ankley et al. 2021). In northern leopard frog (*Rana pipiens*) larvae, exposure to PFOS and PFOA under environmentally relevant conditions caused developmental delays. Impacts in turtles include reduced hatchling emergence success when exposed to long-chain PFCAs, negative correlations between PFAS exposure and body mass, and negative metabolic impacts from PFAS mixtures (Bangma et al. 2019; Wood et al. 2021; Beale et al. 2022).

### Liver enzymes and cholesterol

Activation of the nuclear receptor PPARα is believed to mediate liver toxicity, cholesterol reduction, and contribute to developmental delays in laboratory animals.PPARα is a key regulator of lipid metabolism, cellular growth, and differentiation, and acts as a transcription factor. Exposure to PFOA and PFOS alters gene expression in the PPARα pathway (ATSDR 2021). In rodents, this induces hepatic peroxisome growth and cholesterol deposition in the liver (ATSDR^2021^; Jiang et al. 2015). In police dog and laboratory beagles, PFNA and PFDA were linked to increased cholesterol levels. PFOA and PFOS, in contrast, were related with a decrease in cholesterol levels (You et al. 2022). In cats, Weiss et al.^2021^ found no significant association between cholesterol and PFOA or PFOS. However, total PFAS levels were linked to increased cholesterol (Weiss et al. 202; Brake et al. 2023a, b).

### Prenatal exposure effects

PPARα plays a critical role in rodent embryos. Its activation during gestation has been linked to decreased survival after birth, delayed eye opening, reduced offspring survival, and lower body weights. It's hypothesized that disruptions in lipid metabolism may contribute to these reduced neonatal survival rates and lower body weights(ATSDR 2021). PFAS binds to proteins such as albumin, fatty acid binding proteins, and organic anion transporters. High concentrations of these proteins in blood result in detectable PFAS levels in maternal blood (Ng and Hungerbühler 2013; MacManus-Spencer et al. 2010). This allows PFAS to be transported through maternal circulation to the placental, facilitating fetal exposure during gestation. PFAS’s affinity for maternal blood facilitates them to reach the placenta and enter cord blood, supported by the strong correlation between maternal and cord blood serum (Porpora et al. 2013; Glynn et al. 2012).

### Immunosuppression

Some studies indicate that PFAS exposure reduces T-cell dependent antibody responses and antigen-specific IgM levels in both in vivo and in vitro models (ATSDR 2021; DeWitt et al. 2016). DeWitt et al.^2016^, studied PPARα’s role in immunosuppression and found that while it reduced thymus and spleen weights, it did not affect T-cell–dependent antigen responses or antigen-specific IgM titers (DeWitt et al. 2016). Consequently, the authors suggested that PFAS may impair immunity by targeting B-cell and plasma cell function. Studies on peregrine falcon in the Laurentian Great Lakes (North America) revealed that PFAS exposure impaired immune function and caused physiological effects in nestlings (Sun et al. 2020, 2021).

### Thyroid effects

PFAS have been linked to altered thyroid function in animals, mirroring observations in humans. Two studies identified positive associations between PFAS exposure and hypothyroidism in cats. Bost et al. 2016 reported a strong association between elevated PFOA levels in blood serum and hyperthyroidism, and a weaker link with total PFAS (Bost et al. 2016). The second study found significantly higher levels of PFOA and PFHxS in hyperthyroid cats compared to non-hyperthyroid cats (Wang et al. 2018). Researchers hypothesized that PFAS could alter thyroid function by either elevating thyroid hormone levels or by competing with the binding of triiodothyronine (T3) and thyroxine (T4) to carrier proteins (Chinthakindi et al. 2021; Franko et al. 2012).

### Respiratory effects

Respiratory diseases have been observed in both laboratory animals and domestic cats exposed to PFOA, PFOS, and PFHxS. A study by Bost et al. 2016 found higher concentrations of PFHxS in cats suffering from respiratory disorders compared to healthy controls (Bost et al. 2016). The researchers suggested that PFAS, known to integrate into lipid bilayers, they might disrupt the surface tension of a lung surfactant called dipalmitoylphosphatidylcholine, which is prevalent in cats (Brake et al. 2023a, b). Similarly, laboratory studies reported that inhaling high concentrations of PFOS and PFOA dust led to symptoms such as wheezing and nasal discharge (Averina et al. 2021). Additionally, prolonged exposure to the sodium salt of PFHxA caused reversible degeneration of the nasal olfactory epithelium in rats (Loveless et al. 2009).

In conclusion, PFAS have been linked to a wide array of adverse effects in humans, including metabolic dysfunction, thyroid and cardiovascular disorders, immune suppression, developmental and reproductive issues, and increased cancer risks. Similar effects have been observed in wildlife and companion animals, underscoring the pervasive and systemic nature of PFAS toxicity. Studies across laboratory animals, wildlife, and pets reveal endocrine disruption, metabolic alterations, immune suppression, and developmental toxicity, demonstrating that PFAS impacts are systemic and pervasive, not confined to controlled experimental settings. While species-specific differences in exposure, metabolism, and susceptibility mean that animal data cannot be directly extrapolated to humans, these findings form an essential basis for human health risk assessment.

The growing understanding of the mechanisms through which PFAS disrupt biological systems, together with their environmental persistence, highlights the need to further investigate their long-term impacts and to develop effective strategies to reduce exposure.

## Importance of a One Health approach

### One Health

Is a comprehensive, integrated approach that aims to balance and optimize the health of people, animals, and ecosystems. It recognizes the interconnected nature of human, domestic and wild animal, and environmental health. By linking these three domains, One Health addresses all aspects of disease control, including prevention,detection preparedness, response, and management. This approach ultimately contributes to global health security (World Health Organization 2022). The **One Health** concept emphasizes the necessity to understand how shared environmental factors contribute to the etiology of both human and animal diseases, as well as the degradation of ecosystems. There is increasing interest in applying this approach to identify health risks and inform regulatory and public health responses to chemical hazards such as PFAS (Buttke 2011). Within the One Health framework the concept of Circular Health takes a holistic view of human, animal, and environmental health. It promotes a multidisciplinary approach that complements the biomedical health and incorporates circular economy principles in healthcare. Circular health focuses on sustainable management of health products throughout their life cycle, from production to waste. By leveraging big data and sustainable development tools, it aims to reduce environmental impact and improve healthcare system efficiency. This approach also encourages a shift from reductionist science to a holistic perspective, integrating health and environmental policies to tackle global challenges like pandemics, climate change, and biodiversity loss. This perspective seeks to restore the balance between humans, animals, and the environment, offering solutions that go beyond traditional medical practices and embrace a “circular” philosophy of health (Mantegazza et al. 2023; Capua 2021).

An increasing awareness of the shared environmental risks between animals and humans has led to the greater use of animals as **sentinels** for human exposure to environmental stressors, food safety risks, and other potential health threats (Frazzoli et al. 2015). Sentinel species, which are organisms naturally exposed to pollution and capable of indicating toxicity earlier than humans, provide critical information on biologically relevant toxicity, which can be assessed using biomarkers, quantitative measures of changes in molecular or cellular components, processes, structures, and functions related to exposure to environmental chemicals (Depledge et al. 1995). The ideal sentinel species must meet certain criteria: it should be relatively common across areas and countries, easy to collect, handle, and store in laboratories and exhibit measurable responses to PFAS exposure. Using a widespread species is essential for comparing different sites, as species composition differs across different ecosystems and regions (Van Der Oost et al. 2003). These animals can show signs of harm before humans, making them important indicators of potential environmental health risks (Milnes and Guillette 2008). Wildlife species, particularly freshwater species, have been recognized as sentinels of environmental health and are widely used as a bioindicators of PFAS pollution. Fish living in contaminated waters are exposed to pollutants throughout their lives. Biomarkers in these fish reflect cumulative water quality effects and allow comparisons of water quality across sites over time (Van Der Oost et al. 2003). For example, western mosquitofish (*Gambusia affinis)* is considered an excellent sentinel species for water quality monitoring. Its closerelative, the eastern mosquitofish Poeciliid species (*Gambusia holbrooki)*, has been introduced into over 50 countries and is highly invasive. Both species share life history traits and can hybridize, making them suitable sentinels worldwide. Their widespread distribution, ease of capture, high abundance, and short generation times make them ideal model organisms for evaluating PFAS pollution and overall environmental health. They are already used as bioindicator for other pollutants (Rautenberg et al. 2015; Frankel et al. 2016).

Marine mammals are important sentinel species because many live long lives, reside in coastal areas, feed at high trophic levels, and store toxins in their fat. Many share habitats and food sources with humans, making them effective sentinels of public health risks (Bossart 2011). Dolphins are particularly valuable due to their high site fidelity and long-term residency in certain areas making them important sentinels of the health of coastal marine ecosystems (Wells et al. 2004; Díaz López 2019; Bilela et al.^2023^). In New Mexico vertebrates, including birds, showed extremely high PFAS levels contaminated areas. Birds from the Holloman Air Force Base had tissue PFAS levels over 30 times higher than control sites. PFOS was the predominant compound, showing liver concentrations that averaged above 10,000 ng/g wet weight (ww) in birds and mammals, and reaching 97,000 ng/g ww in a single case. PFHxS also showed liver levels in the thousands of ng/g ww in aquatic birds. These findings indicate significant bioaccumulation in tissues, particularly in the liver, linked to multiple exposure pathways such as ingestion of surface water, sediments, and contaminated preys (Witt et al. 2024).

Pets can also serve as sentinels for human exposure., A study comparing cats and humans in the same location and period found similar PFAS levels (15.8 ng/mL for cats vs. 14.3 ng/mL for humans) (Wang et al. 2018). **Horses**, while less studied, are valuablesentinels. They live near humans homes but spend much of their lives outdoor, reflecting both human and local wildlife exposure. A study measuring PFAS levels in the blood of dogs and horses underlines the potential of companion animals and livestock as sentinels for environmental exposure, highlighting differences in exposure indoor and outdoor environments (Rock et al. 2023). PFAS levels in pets serum reflects their primary living environment and water sources. Indoor dogs and cats tend to have higher PFAS than outdoors or feral ones (Rock et al. 2023). The widespread presence of domestic animals, along with the accessible health diagnostic methods and shared diseases with humans, highlights the importance of a One Health strategy to investigate environmental exposures and their effects on humanand animal health.

## Regulatory aspects

The widespread use of PFAS, coupled with their persistence in the environment, has led to significant contamination of both ecosystems and food sources. This contamination arises mainly from bioaccumulation in aquatic and terrestrial food chains. Diet is the major source of PFAS exposure. Food contact materials containing PFAS also contribute to human contact (European Commission 2022). Consequently, the Stockholm Convention introduced regulations in 2009 to control PFOS and its precursors (Liu et al. 2022b). Over time, these regulatory measures have been extended to include PFOA, PFHxS, their salts, and related compounds. This has driven an increased focus on identifying substitutes for traditional long-chain PFAS, such as short-chain homologs and novel fluorinated alternatives (Feng et al. 2019).

In 2018, the Organization for Economic Co-operation and Development (OECD) (OECD 2018) updated the 2007 PFAS list and created a comprehensive global database providing detailed information on PFAS. This database includes data on their uses, chemical properties, environmental distribution, and potential health impacts, serving as a valuable resource for environmental policy, public health regulation, and scientific research. The project involved broad international collaboration, collecting data from OECD member countries and other organizations. Its goal was to enhance global understanding of the risks associated with PFAS. The updated database has improved global PFAS monitoring, incorporating new data from countries that previously lacked the capacity or had not reported sufficient information.

In summary, the initiative improved the global understanding of PFAS.It also strengthened the capacity to monitor, manage, and regulate these chemicals in the context of environmental and public health challenges.

Legislatures worldwide are responding to the risks PFAS pose by implementing measures to control their use. In Europe and the USA, authorities have recently proposed various legislative actions aimed at reducing or eliminating PFAS in consumer products.

**Fig. 3:**
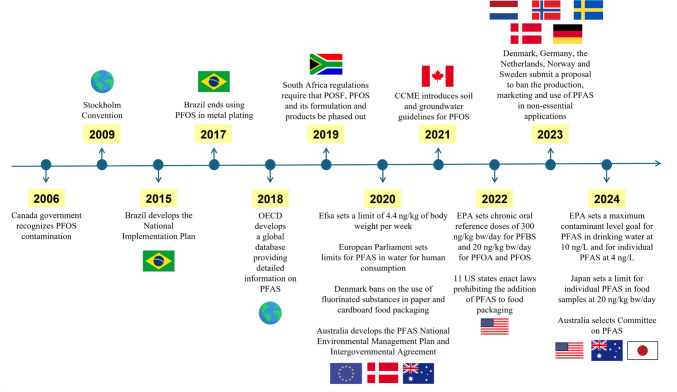
Timeline of per- and polyfluoroalkyl substances legislation

This section provides an overview of PFAS regulations for each continent, while Fig. 3 presents a timeline of PFAS legislation.

## Europe

In June 2020, the Danish Ministry of Environment and Food introduced a ban on the use of fluorinated substances in paper and cardboard food packaging, unless a functional barrier to prevent their migration into food (Danish veterinary and food administration 2020). The European Union's REACH regulation (Registration, Evaluation, Authorization, and Restriction of Chemicals) also restricts the use, manufacture, import, and export of specific PFAS (Chen et al. 2024). In 2023, five EU Member States (Denmark, Germany, the Netherlands, Norway, and Sweden) proposed to the European Chemical Agency (ECHA) a ban on the production, marketing and use of PFAS in non-essential applications (Jovanović et al. 2024). EFSA has recommended a limit on combined exposure to four PFAS (PFOA, PFNA, PFOS and PFHxS) in food, setting a limit of 4.4 ng/kg of body weight per week. Packaging materials were found to contribute between 17.5 and 45.9 µg/kg (Jovanović et al. 2024; Muncke et al. 2023). Additionally, to ensure the quality of water for human consumption, Directive (EU) 2020/2184, issued by the European Parliament and Council of 16 December 2020, sets limits for PFAS (OECD 2021). These include a threshold of 500 ng/L for the ‘*PFAS Total*’ parameter and 100 ng/L for the ‘*Sum of PFAS*’ parameter, covering a list of 20 PFAS (European Commission 2024). Member states must comply with these limits by January 12, 2026 (European Commission 2009).However, they may adopt stricter thresholds or include additional parameters in their national legislation (European Commission 2024). Continuous restrictions on the manufacturing, application, and disposal of PFAS-containing products are expected to mitigate the overall risks posed by these chemicals (Schiavone and Portesi 2023).

Regarding emerging PFAS, some regulatory measures exist, such as the classification of HFPO-DA and PFBS as “substances of very high concern” under REACH (Frigerio et al. 2022). However, many novel PFAS remain poorly studied, and their toxicological profiles and environmental behaviors are still unclear (Brase et al. 2021; Frigerio et al. 2022; Manojkumar et al. 2023).

### America

#### United States

In the USA, the Environmental Protection Agency (EPA) has set chronic oral reference doses of 300 ng/kg body weight per day for PFBS and 20 ng/kg body weight per day for PFOA and PFOS (EPA 2022). In 2018, the US Food and Drug Administration (FDA) reported that wastewater from typical paper mills released approximately 40 to 100 kg of PFAS per day (Simonetti et al. 2024). By 2022, 11 US states had enacted laws prohibiting the addition of PFAS to food packaging (Hassan et al. 2024). Additionally, the EPA has set the maximum contaminant level goal for PFAS in drinking water at 10 ng/L, with the maximum contaminant level for individual PFAS set at 4 ng/L (EPA 2024a, 2024b).

### Canada

In 2006, the Canadian federal government identified perfluorooctane sulfonate (PFOS), its salts, and related compounds as environmental risks due to their potential to harm ecosystems and biodiversity. At that time, a human health assessment determined that PFOS exposure levels were below thresholds of concern. By 2012, Canada updated its list of prohibited toxic substances to include PFOS and related chemicals.

In 2016, Health Canada expanded its drinking water screening to encompass seven additional per- and polyfluoroalkyl substances (PFAS) beyond PFOS and PFOA. A year later, soil screening regulations were introduced. Environment and Climate Change Canada (ECCC) also issued federal environmental quality guidelines for PFOS to mitigate risks.

By 2021, the Canadian Council of Ministers of the Environment (CCME) established comprehensive soil and groundwater guidelines for PFOS, integrating standards from Health Canada and ECCC (Hains 2023; Environment and Climate Change Canada 2023).

### Brazil

In 2015, Brazil developed a series of regulation actions focused on specific substances, such as PFOS and its related compounds, as outlined in the National Implementation Plan (NIP-Brazil-2015). To date, Brazil remains the only country in Latin America with an ambitious national proposal for broad PFAS control. However, some proposed activities remain incomplete or have yet to be initiated. The country has identified limited uses of PFOS, mainly in sulfluramid-based insecticides and as a mist suppressant in metal plating. However, its authorized use in Brazil exceeds the exemptions outlined in international agreements, leading to regulatory adjustments. The Brazilian Health Regulatory Agency (ANVISA) has re-evaluated the registration of household pesticides containing sulfluramid, implementing a phase-out period for existing stocks. Additionally, efforts are underway to identify alternative substances to replace sulfluramid in both agriculture and domestic applications.

Since 2017, Brazil has discontinued PFOS use in metal plating, though it continues to import POSF (perfluorooctane sulfonyl fluoride) for pesticide production. Even so, stakeholder engagement in regulation remains low, making it difficult to inventory and monitor these substances. Additionally, Brazil is working to update its National Implementation Plan to strengthen PFAS control, promoting the use of specific customs codes for tracking imports and exports and improving environmental management strategies (Barbosa Machado Torres et al. 2022). There are ongoing studies to assess the environmental impact of PFOS applications, as well as initiatives to explore non-chemical alternatives. Despite these measures, Brazil still lacks comprehensive regulations covering the broader range of PFAS chemicals, highlighting the need for further legislative advancements and monitoring strategies (Ministry of the environment 2015).

### Asia

Some of the major Asian countries, including China, Japan, and South Korea, have begun to implement stricter regulations on products containing PFAS (Schiavone and Portesi 2023). In 2024, Japan’s Food Safety Commission announced a daily intake limit for PFAS, setting the tolerable limit for individual PFAS in food samples at 20 ng/kg body weight per day (Otake 2024).

### Africa

#### South Africa

South Africa's legislation on PFAS is still evolving. Currently, regulations mandate that POSF, PFOS, and its formulation and products, be phased out by December 2021 (Department of environmental affairs 2019). Although the risks associated with persistent contaminants like PFAS have been recognized, South Africa has yet to establish comprehensive regulations to restrict their use and monitor their environmental impact. The implementation of stricter management and control strategies remains a critical challenge for the future (Water Research Commission 2020).

### Oceania

#### Australia

Australia adopted a PFAS contamination response protocol, implemented a PFAS national environmental management plan, and established guidelines to support government agencies responding to PFAS contamination. The PFAS National Environmental Management Plan (NEMP) (Heads of EPA Australia and New Zealand 2020) provides a practical, risk-based framework for regulating PFAS-contaminated materials and sites. Additionally, the Intergovernmental Agreement on a National Framework for Responding to PFAS Contamination offers specific guidance on actions for managing contaminated sites (Council of Australian Governments 2020). On 22 August 2024, the Senate appointed a Select Committee, to investigate the extent of PFAS contamination, its regulation, and management. This committee is expected to present its final report by 5 August 2025 (Parliament of Australia 2024).

Despite growing awareness, many regions still lack PFAS regulations. This hinders elimination efforts, since their presence crosses national borders. The shift to short-chain PFAS reflects regulatory compliance rather than a true reduction in environmental or health risks. This raises critical questions about the effectiveness of regulatory frameworks in promoting sustainable alternatives and preventing the spread of equally hazardous substitutes. While some nations regulate only the most well-known PFAS, emerging variants often remain unregulated, allowing their continued production and distribution. In other regions, no PFAS regulations exist, further complicating global elimination efforts. Countries like Italy have begun monitoring specific PFAS, such as C6O4 in major water systems, but many areas of the world still lack effective control mechanisms (Bernardini et al. 2021; ARPA Lombardia 2024; ARPA Veneto 2023). Their global dispersal, lack of regulations, and limited treatment technologies highlight the urgent need for international research to address persistence and health risks.

## Relevance of methods for monitoring and research

Gas (GC) and liquid chromatography (LC) coupled with tandem mass spectrometry (MS/MS) are standard analytical techniques for PFAS detection (Rodriguez et al. 2020). These techniques provide high levels of selectivity and sensitivity and can measure substances at very low concentrations (Chandrasekaran et al. 2024). For PFAS, LC-MS/MS methods developed with electrospray ionization (ESI) in negative mode are suitable for many anionic compounds. However, comprehensive PFAS characterization, requires alternative instrumental conditions to analyze zwitterionic and cationic PFAS, as well as gas GC-MS/MS for volatile PFAS (Szabo et al. 2024).

Despite these advancements, established PFAS analysis methods primarily rely on LC-MS/MS, due to sensitivity and ability to detect multiple compounds simultaneously. Over the years, this technique has been refined to analyze over than 50 PFAS in a single analytical run (Coggan et al. 2019). Conventional reverse-phase separation using stationary phase columns remains the most common approach. To enhance chromatographic performance, polar functionalized C18 alkyl chain columns were introduced (Kennedy et al. 2021). These columns better retain polar compounds, particularly the shorter-chain PFAS eluting early in the chromatographic run (Schiavone and Portesi 2023).

As regulatory bodies establish permissible PFAS levels, accurate detection becomes increasingly critical. Consequently, analytical methods are continually refined to achieve lower detection limits, detect a greater number of analytes, and identify previously uncharacterized PFAS (Brase et al. 2021; Szabo et al. 2023).

However, PFAS analysis is fraught with challenges, particularly background contamination. The ubiquitous presence of PFAS in laboratory supplies and reagents can result in false detections. Additionally, PFAS tend to adhere to container walls, forming a residue that complicates accurate measurements. As a result, extreme care is required during sample preparation to avoid contamination and false positive results. In PFAS analysis, polypropylene materials are preferred to glass, since PFAS tend to adhere to glass surfaces and compromise accuracy. Polypropylene, being less reactive, reduces the risk of contamination during sample collection and analysis (Shoemaker and Tettenhorst 2020). Even with meticulous sample preparation, analytical instruments may introduce PFAS contamination from internal components. To reduce this risk, PFAS-free materials and isolator columns are used to trap residual PFAS and separate them from the sample. However, complete removal of all PFAS from an analytical system remains challenging due to the diverse range of PFAS compounds (Brase et al. 2021). Moreover, sampling from air, water, soil, and biological tissues requires standardized procedures to avoid contamination and ensure accuracy (Brase et al. 2021).

**Fig. 4:**
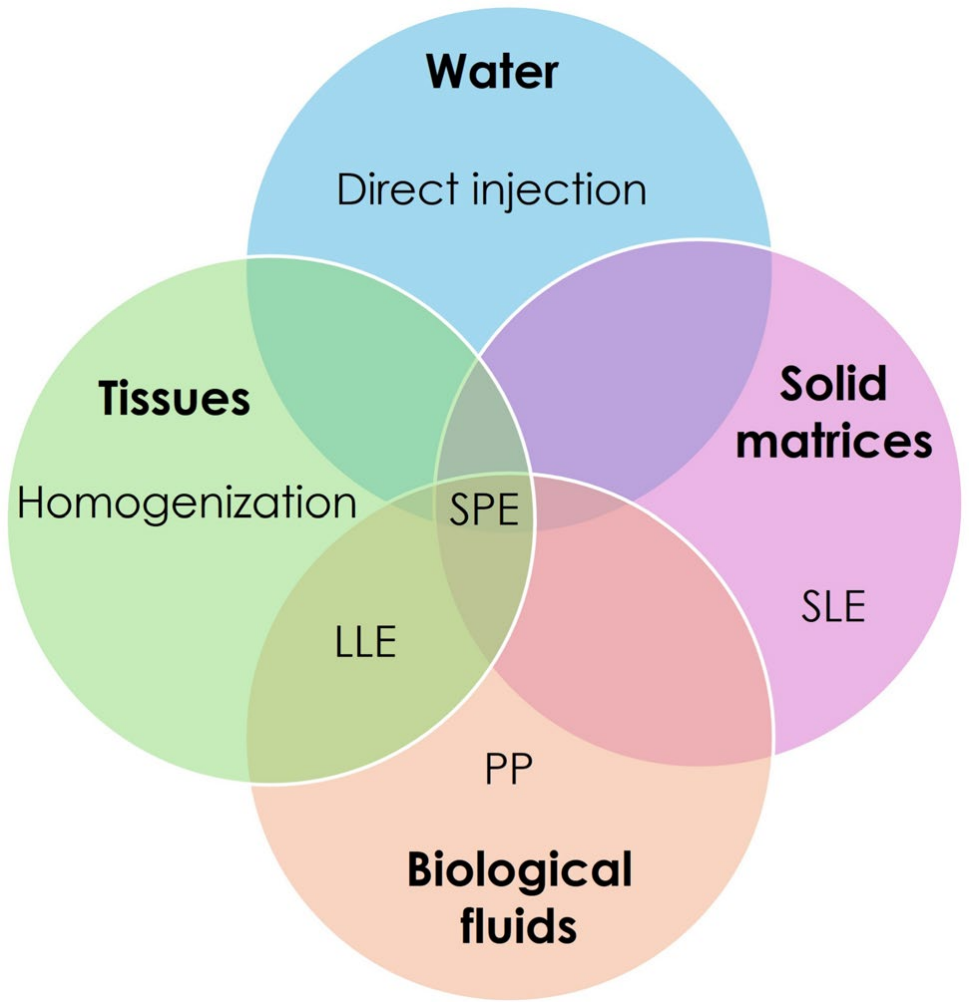
Sample preparation approaches for various matrices, including water, biological fluids, and solid tissues or substrates. The techniques shown — Solid–liquid extraction (SLE), Solid-phase extraction (SPE), Protein precipitation (PP), and Liquid–liquid extraction (LLE) — are fundamental steps prior to liquid chromatography tandem mass spectrometry (LC-MS/MS) analysis

Another challenge is balancing the detection of legacy PFAS with emerging compounds. Monitoring groups is essential for comprehensive exposure assessment (Frigerio et al. 2022). Analytical methods often show higher sensitivity for well-known, stable PFAS, but are less effective for newer, less studied variants with differing chemical properties. Achieving comprehensive detection across the PFAS spectrum remains a key focus for analytical development (Coggan et al. 2019). To address these challenges, tailored liquid chromatography-tandem mass spectrometry techniques have been developed for different PFAS classes and sample matrices (Frigerio et al. 2022). The United States Environmental Protection Agency (USEPA) has recently proposed protocols for analyzing PFAS in complex matrices, including biological tissues. The LC-MS/MS combined with isotopically labelled standards is utilized for these analyses. The ability to measure PFAS in various matrices is functional for understanding their impacts on environment and human health (Chandrasekaran et al. 2024). Environmental studiesrequire multiple methods due to the variety of sample types. These include biological matrices (e.g., blood, serum, liver) and environmental samples (e.g., water, soil, sludge). Sample preparation is crucial for PFAS analysis, to enhance selectivity, sensitivity, and analyte purification (Shen et al. 2024). Each sample type demands customized extraction and cleanup processes, illustrated in Fig. 4 (Clarke 2024). For liquid matrices, such as water, direct injection can be used for clean samples (Pietropoli et al. 2024; EPA 2021), while Solid Phase Extraction (SPE) is preferred when analyte concentration or purification is needed (EPA 2024c, 2019; Getzinger and Ferguson 2021). Solid matrices, including soil, sediment and sludge, often require solid–liquid extraction (SLE) or SPE to isolate target compounds (Waters 2023; EPA 2024c). Biological tissues are homogenized to break down the sample structure, followed by SPE or liquid–liquid extraction (LLE) for purification (EPA 2024c; Shen et al. 2024; Q. Wang et al. 2021). Biological fluids, such as plasma, serum, or urine, require protein precipitation (PP) to remove interfering proteins.After this step, SPE or LLE is applied to extract the analytes (Shen et al. 2024; Al Amin et al. 2020; Szabo et al. 2022; Da Silva et al. 2020; Yao et al. 2020; Shi et al. 2020). The choice of method depends on the sample type, the target analytes, and the analytical requirements.

Robust analytical methods are essential for establishing safety limits, monitoring contamination trends, identifying exposure sources, and understanding the long-term health effects of PFAS.

## Remediation strategies

Although challenging due to PFAS chemical stability and mobility, effective remediation strategies are essential to mitigate their impact. Contaminated media can be treated and remediated at its origin (e.g., industrial wastewater), at secondary concentration points (e.g., drinking water facilities or landfill leachate), or within widespread environmental areas (e.g., groundwater) (Evich et al. 2022). Strategies vary depending on the environmental matrix affected, and the following is a synthesis of the methods, including advanced techniques, and challenges associated with their application.

## Water treatment

Granular Activated Carbon (GAC) and Ion-Exchange Resins are commonly used for adsorbing PFAS, particularly long-chain variants (Crone et al. 2019). These methods are effective for treating drinking water. However, their efficiency decreases with short-chain PFAS due to their higher mobility and lower adsorptive affinity. Granular Activated Carbon must be managed carefully, as it can become a secondary source of contamination if not properly treated. PFAS and other contaminants adsorbed onto the GAC can desorb back into the environment if the spent GAC is exposed to conditions that reduce adsorption affinity, such as changes in pH, temperature, or the presence of competing substances. This can lead to secondary contamination of water or soil during disposal or storage. Once PFAS are adsorbed onto GAC, they must be carefully destroyed (e.g., incineration at high temperatures) to prevent their re-entry into the environment (Evich et al. 2022).

## Groundwater remediation

There are several approaches for groundwater remediation, including both containment techniques, such as pump-and-treat technologies or barrier methods and decontaminated strategies like in situ foam fractionation (Held and Reinhard 2020).

Pump-and-treat systems are widely used to contain and treat PFAS plumes. The process involves two steps: extraction and treatment. First, contaminated groundwater is pumped to the surface using wells strategically placed to capture the plume and prevent further migration through a controlled hydraulic barrier. The extracted groundwater is then treated to remove PFAS and other contaminants. Commontreatment methods include adsorption onto granular activated carbon (GAC), ion-exchange resins, or membrane-based filtration systems like reverse osmosis. After treatment, the water is either discharged back into the environment (e.g., a river or aquifer) or reused, ensuring compliance with environmental standards. However, this technique has some limitations. It does not destroy PFAS but only transfer them from groundwater to another medium (e.g., adsorbent material), which requires careful disposal. While effective in controlling plume migration, it is often slow in achieving full remediation, particularly for large or diffuse plumes (Evich et al. 2022; Held and Reinhard 2020).

The funnel-and-gate system is a groundwater remediation technology designed to control and treat PFAS plumes. The “funnel” component consists of impermeable barriers, such as sheet piling or slurry walls, installed in the subsurface. These barriers direct groundwater flow toward the “gate,” a permeable zone containing reactive or adsorptive materials such asactivated carbon or ion-exchange resins. As the contaminated groundwater passes through the gate, the system concentrates and treates the contaminants efficiently. Although this system is effective for containing plumes and minimizing the spread of PFAS, its application is limited to relatively stable plumes and requires significant design and maintenance, particularly in ensuring the gate’s materials remain effective over time (Evich et al. 2022; Held and Reinhard 2020).

In situ foam fractionation is an emerging remediation technology for groundwater treatment exploiting the surfactant-like properties of PFAS, which accumulate at air-water interfaces. Air or gas is injected into the contaminated water, creating bubbles that adsorb PFAS due to their hydrophobic and hydrophilic properties. The bubbles rise to the surface forming a foam layer enriched with PFAS, which is then collected for further treatment or disposal. Although energy-efficient and suitable for localized areas with high PFAS concentrations, its large-scale application is limited. High costs, inefficiency in treating massive plumes, and the need for specialized infrastructures constrains its use. Additionally, the process may not completely eliminate PFAS (Held and Reinhard 2020).

## Soil remediation

Soil remediation for PFAS contamination involves strategies to address the mobility, persistence, and toxicity of these substances. The main approaches include:

### Excavation and landfilling

This method involves the removal of contaminated soil and disposal in secure landfills. While effective at isolating PFAS, it has some limitations such as high transportation and disposal costs, regulatory constraints on landfill capacity, and the fact that it merely relocates the problem rather than eliminating it (Brunn et al. 2023).

### Soil sorption enhancement

Long-chain PFAS are known to bind to soil organic matter, particularly in soils with high organic content. Techniques that mix soil with binding agents, such as cement, can reduce PFAS mobility by enhancing the soil's binding capacity. However, this does not address the persistence of PFAS, and while it prevents leaching into groundwater, the long-term stability of the treated material requires further investigation.

### Soil flushing

Chemical agents or surfactants are injected into the soil to mobilize PFAS, which are then recovered through extraction wells. This reduces the need for excavation but can generate secondary wastewater, which requires treatment (Held and Reinhard 2020; Brunn et al. 2023).

### Foam fractionation

Similar to groundwater remediation, this technique exploits the surfactant-lie properties of PFAS. Air bubbles capture the substances at the air-water interface, forming a foam that is collected for treatment or disposal. It shows potential for localized treatment, but exhibits high operational costs and is not yet viable for large-scale or deep contamination scenarios (Held and Reinhard 2020; Brunn et al. 2023).

### Thermal treatments

Incineration at very high temperatures (≥ 1000 °C) can break the C-F bonds, destroying PFAS and converting theme mineral components. Ideally, this process produces gases like HF, NOx, SOx, and CO2, which are managed using standard air pollution controls. However, complete PFAS conversion to HF and non-fluorinated substances requires maintaining high temperatures for a prolonged period. Heavily fluorinated compounds needeven longer times and higher temperatures (Khan et al. 2020; Rayne and Forest 2009). Thermal processes can destroy of both long-chain and short-chain vPFAS. Hoewever, they require specialized incinerators, high energy input, and incur significant operational costs. In addition, these processes have not been demonstrated at scale, where inefficiencies can reduce performance (Evich et al. 2022).

### Preventive and regulatory approaches

Introducing barriers in agricultural systems to limit PFAS movement through runoff and leaching, and controlling PFAS inputs through biosolid testing and treatment prior to land application, are essential for preventing new contamination (Held and Reinhard 2020; Verley et al. 2024). These strategies highlight the need formultifaceted approaches combining physical, chemical, and biological methods. Research gaps, particularly for short-chain and emerging PFAS, remain a barrier to effective remediation, while energy demands and potential hazardous byproducts further complicate widespread adoption (Held and Reinhard 2020; Brunn et al. 2023).

Overall, these challenges underscore the importance of both advancing remediation technologies and implementing preventive measures, including stricter regulation and reduction of PFAS release, to protect environmental, human, and animal health.

## Conclusion

In conclusion, this review synthesizes the existing literature on PFAS, emphasizing their role as one of the most significant environmental and public health challenges. These substances, marked by their extreme persistence and bioaccumulation potential, pose significant threats to both human and animal health. Their resistance to degradation and ability to accumulate in the biological tissues amplify their long-term impacts, which remain poorly understood, especially in animals. The growing evidence of their harmful effects, including hormonal, immunological, and carcinogenic disorders, has prompted global calls for more cohesive and interdisciplinary responses. One such approach, the “One Health” model, emphasizes the interconnectedness of human, animal, and environmental health, highlighting the role of animals as sentinels for environmental contamination and exposure. While advancements in analytical techniques, particularly LC-MS/MS, have greatly enhanced the ability to monitor PFAS at trace levels and across diverse matrices, significant challenges still persist. These methods have improved the precision and comprehensiveness of environmental screening, yet the complexity of PFAS mixtures and their prevalence in minute concentrations complicate detection and control efforts. The emergence of novel, less characterized PFAS compounds exacerbates this challenge. Many of these newer substances remain unregulated, and data on their toxicological profiles, bioaccumulation tendencies, and environmental persistence are sparse.

This knowledge gap is particularly concerning given the rapid pace of industrial innovation and substitution of regulated PFAS with chemically similar alternatives. Without timely identification and regulatory oversight of these emerging PFAS, the potential for unanticipated long-term harm increases significantly.

Moving forward, the scientific community must prioritize research into the impacts of PFAS on animals, whose health can serve as an early warning system for broader ecological and human health risks. This entails a concerted global effort to refine analytical methodologies, accelerate toxicological evaluations, and implement stricter regulations that encompass both legacy and emerging PFAS.

## Data Availability

Data sharing is not applicable to this article as no datasets were generated or analyzed during the current study.
